# Optimizing Wide Band Gap Cu(In,Ga)Se_2_ Solar Cell Performance: Investigating the Impact of “Cliff” and “Spike” Heterostructures

**DOI:** 10.3390/ma17215199

**Published:** 2024-10-25

**Authors:** Shiqing Cheng, Hongmei Liu, Qiaowen Lin

**Affiliations:** College of Physics and Electronic Science, Shanxi Datong University, Datong 037009, China; lhm9898@163.com (H.L.); lqw16888@163.com (Q.L.)

**Keywords:** wide band gap CIGS, thin film, interface structure, recombination

## Abstract

In recent years, the efficiency of high-efficiency Cu(In,Ga)Se_2_ (CIGS) solar cells has been significantly improved, particularly for narrow-gap types. One of the key reasons for the enhancement of narrow-gap device performance is the formation of the “Spike” structure at the CdS/CIGS heterojunction interface. Wide-gap CIGS solar cells excel in modular production but lag behind in efficiency compared to narrow-gap cells. Some studies suggest that the “Cliff” structure at the heterojunction of wide-gap CIGS solar cells may be one of the factors contributing to this decreased efficiency. This paper utilizes the SCAPS software, grounded in the theories of semiconductor physics and photovoltaic effects, to conduct an in-depth analysis of the impact of “Cliff” and “Spike” heterojunction structures on the performance of wide band gap CIGS solar cells through numerical simulation methods. The aim is to verify whether the “Spike” structure is also advantageous for enhancing wide-gap CIGS device performance. The simulation results show that the “Spike” structure is beneficial for reducing interfacial recombination, thereby enhancing the *V*_OC_ of wide-gap cells. However, an electronic transport barrier may form at the heterojunction interface, resulting in a decrease in *J*_SC_ and *FF*, which subsequently reduces device efficiency. The optimal heterojunction structure should exhibit a reduced “Cliff” degree, which can facilitate the reduction of interfacial recombination while simultaneously preventing the formation of an electronic barrier, ultimately enhancing both *V*_OC_ and device performance.

## 1. Introduction

In recent years, CIGS thin-film solar cells have gained widespread application in large-scale industrial production. Ideally, terrestrial solar cells require a wide band gap of at least 1.5 eV to align with the solar spectrum [1,2,3,4,5,6,7,8,9,10,11]. Consequently, for CIGS solar cells to achieve such a band gap, their absorber must possess a high GGI (Ga/(In + Ga) ≥ 0.8). In addition to their impressive ideal efficiency, CIGS solar cells with such a wide band gap also exhibit high *V*_OC_, low *J*_SC_, and a superior temperature coefficient. These attributes are beneficial for minimizing losses arising from interconnecting individual cells, thereby improving their suitability for large-area modules. Nevertheless, in practical applications, CIGS solar cells with a narrower band gap (1.15~1.2 eV) and a GGI of 0.3~0.4 have proven to be the most efficient [4,12,13,14]. The main reasons for the low performance of CIGS solar cells with wide band gap are as follows: (1) insufficient bending of the energy band on the surface of the absorber and weak interface inversion lead to increased interfacial recombination and a decreased *V*_OC_ of the device [1]; (2) a high defect density at the heterojunction interface will also lead to increased interfacial recombination, resulting in a decrease in the *V*_OC_ of the device [1]; (3) a short minority carrier lifetime results in weak carrier collection and decreases device performance [15]; (4) The CdS/CGS heterojunction being in a “Cliff” structure may results in increased interfacial recombination, causing a decrease in the *V*_OC_ of the device [16,17]. It is well known that, for narrow-band CIGS solar cells, the “Spike” structure of the CdS/CIGS heterostructure is beneficial to the improvement of device performance [16,17]. Thus, is it also beneficial to adopt a “Spike” structure between the buffer layer and the CIGS layer to improve the efficiency of wide-gap CIGS solar cells? K. Nakashima et al. believe that the “Spike” structure can assist in reducing interfacial recombination, thus improving the device’s performance [18]. K. T. Ramakrishna Reddy and S. Sharbati et al. used the adjustable band gap films Cd_x_Zn_1−x_S and Zn(O, S) as buffer layers, respectively, and their experimental results suggest that the “Spike” structure does not help to improve the performance of wide-gap CGS solar cells [19,20]. Fredrik Larsson et al. used the adjustable band gap buffer layer Zn_1−x_Sn_x_O_y_ to match with the wide band gap CIGS layer, which greatly improved the *V*_OC_ of CGS devices and thereby improved the performance of the devices [21]. However, the heterostructure between Zn_1−x_Sn_x_O_y_ layers with tunable band gap and wide band gap CIGS layers has not been clearly indicated. Although some researchers have investigated this issue, whether the “Spike” structure is beneficial to improving wide-gap CIGS device performance remains controversial. This article takes the wide band gap CGS solar cell as an example and utilizes the SCAPS simulation software (3.3.05) to conduct a detailed analysis. In particular, the effect of CdS/CGS heterojunction with “Spike” and “Cliff” structures on the interface and bulk recombination is studied [22]. The purpose of the research above is to obtain the optimal band gap mismatch of heterojunction that can improve the performance of wide band gap CGS solar cells. According to the research, we conclude that the “Spike” structure of the CdS/CGS heterostructure is not beneficial to the improvement of the performance of CGS solar cell devices. The weak “Cliff” structure of the CdS/CGS heterojunction is the most favorable for the improvement of device performance.

## 2. Methods

**Figure 1:**
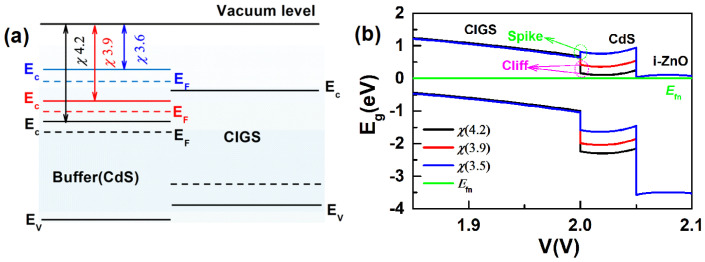
(**a**) Band structure of a CdS/CGS heterojunction with different electron affinities (*χ*) of the CdS layer; (**b**) *χ* = 4.2, 3.9 are the “Cliff “structure, *χ* = 3.5 is the “Spike” structure.

In this paper, the SCAPS (3.3.05) simulation software is used to analyze the impact of changes in the heterostructure on the performance of CGS devices [22]. The aim of this work is to investigate whether the “Spike” structure is necessary for wide band gap CGS solar cells. We conduct the research using the CdS buffer layer as an example, primarily by varying its electron affinity to create a CdS/CGS heterojunction in either a “Cliff” or “Spike” structure, as shown in Figure 1a,b. In the simulation process, the doping concentration (*N*_D_) of the CdS film is set to 10^17^ cm^−3^, with a thickness of 50 nm. The doping concentration (*N*_A_) of the CGS absorber layer is 10^16^ cm^−3^. The interface defects between the absorber layer and the buffer layer are assumed to be neutral, with an electron capture cross-section and a hole capture cross-section of 10^−13^ cm^−2^ distributed in a single, and a total defect density of 10^12^ cm^−2^ [23]. The energy level (*E*_d_) of the defects is set at 0.8 eV above the *E*_V_ [24] and the interface recombination rate for electrons and holes (*S*_n_/*S*_p_) is 10^6^/s [25]. The parameters of the other layers are shown in Table 1. The detailed simulation process of SCAPS (3.3.05) is displayed in the Supplementary File.

## 3. Results

**Figure 2:**
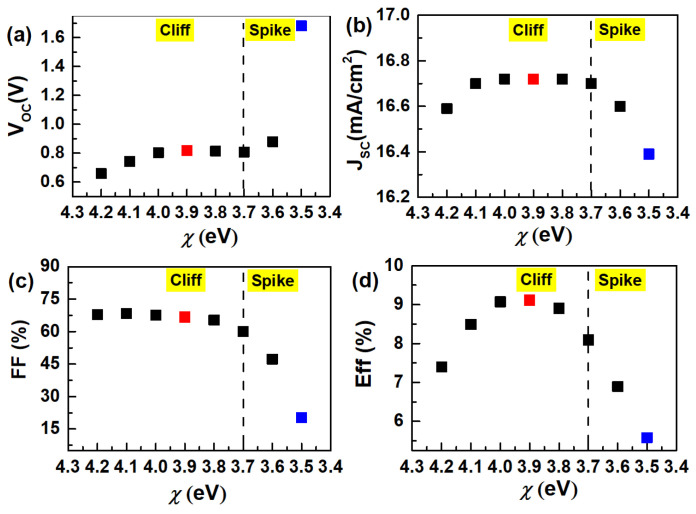
Electrical parameters of CGS devices when the CdS/CIGS heterojunction is in “Cliff” (the left of dashed line) and “Spike” (the right of dashed line) structures: (**a**) *V*_OC_, (**b**) *J*_SC_, (**c**) *FF*, (**d**) *E*_ff_. The black squares on the graph represent the device performance at different *χ* values of the CdS layer. Among them, red squares highlight the highest performance at *χ* = 3.9 eV, and blue squares indicate the lowest performance at *χ* = 3.5 eV.

By adjusting the electron affinity (*χ*) of the CdS layer, the band gap mismatch of the CdS/CGS heterojunction can be altered, thereby changing its band structure. As shown in Figure 1, as the electron affinity of the CdS buffer layer decreases (from 4.2 eV to 3.5 eV), the CGS/CdS heterojunction gradually transforms from a “Cliff” structure to a “Spike” structure. Figure 2 demonstrates that during the transition of the CdS/CIGS heterojunction from the “Cliff” structure to the “Spike” structure, the *V*_OC_ of the CGS device gradually increases, while the *J*_SC_ first increases and then decreases. The *FF* remains relatively unchanged when the heterojunction is in the “Cliff” structure, but rapidly decreases as the “Spike” effect strengthens. The efficiency (*E*_ff_) of the CGS device first increases and then decreases during the transition from the “Cliff” structure to the “Spike” structure. The *E*_ff_ of the CGS device reaches its peak when the electron affinity (*χ*) of the CdS layer is around 3.9 eV (Cliff), mainly due to the increase in *V*_OC_ and *J*_SC_ (Figure 2a–c). However, when the electron affinity of CdS is around 3.5 eV (Spike), the performance of the CGS device significantly decreases. The main reasons for this decline are the decreases in *J*_SC_ and *FF* (Figure 2a–c). To understand the impact of changes in the band structure of the CGS/CdS heterojunction on the performance of the CGS device, we conducted a detailed analysis of its recombination behavior.

### 3.1. The Impact of “Cliff” Structure in CdS/CGS Heterojunction on Device Performance

**Figure 3:**
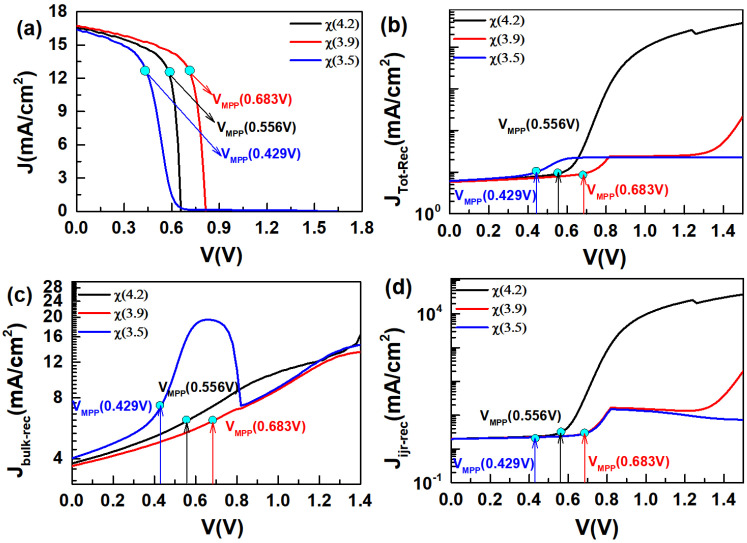
(**a**) The *J-V* curve of solar cells with the CdS electron affinity (*χ*), (*χ*(4.2), black), (*χ*(3.9), red) and (*χ*(3.5), blue). The curve of (**b**) Jtotal-Rer-V, (**c**) Jbulk-V, (**d**) Jifr-V representing the recombination current density of total recombination, bulk recombination, and interfacial recombination, respectively.

When the electron affinity (*χ*) of the CdS layer decreases from 4.2 eV to 3.9 eV, the CdS/CGS heterojunction remains in the “Cliff” structure (Figure 1b). At this point, the V_OC_ and J_SC_ of the device increase, while the FF remains relatively unchanged, resulting in optimal device performance (Figure 2a–d). Since the total electric field of the heterojunction weakens under high bias voltages, the role of recombination becomes more obvious. In this study, we investigate the impact of heterojunction recombination on the V_OC_ of the device at a high bias voltage of 1.4 V < E_g_ (1.68 eV). It can be observed from Figure 3a–d that when the electron affinity of the CdS layer decreases to 3.9 eV, the total recombination (J_Tot-Rec_) (1.4 V) decreases, which may be the primary reason for the increase in V_OC_. The decrease in (J_Tot-Rec_) (1.4 V) is primarily influenced by the reduction in interfacial recombination (J_jir-rec_) (1.4 V), as shown in Figure 3b–d. Therefore, increasing the electron affinity to 3.9 eV (Cliff) helps reduce the interfacial recombination under high bias voltages, thereby enhancing the V_OC_ of the device. This is in line with the results of the Nyquist plot, in which the recombination resistance (R_p_) is increased (Figure S1) [28,29]. The impact of changes in the heterojunction structure on interfacial recombination can also be explained by the distribution of electrons and holes at the interface (in the dark state) as shown in Table 2. The influence of electrons and holes at the heterojunction interface on the interfacial recombination rate (R) is described by Equation (1) [30].

(1) $$R=\frac{n^{if}p_{a}^{if}-n_{i}^{2}}{S_{p}^{-1}(n^{if}+n^{*})+S_{n}^{-1}(p_{a}^{if}+P^{*})}$$

where $n^{if}$ is the electron concentration at the interface, $p_{a}^{if}$ is the hole concentration on the CGS side at the interface, S_p_ and S_n_ are the recombination rates of holes and electrons at the interface, respectively, n* and p* represent the defect state concentrations of electrons and holes at the interface, E_d_ is the defect energy level, E_C_ and E_V_ are the conduction band and valence band energies, respectively, and N_C_ and N_V_ represent the effective state densities of the conduction band and valence band, respectively.

Based on the data in Table 1 and Table 2 and Equation (1), it can be concluded that $n^{if}\gg n^{*}$, $p_{a}^{if}\gg p^{*}$, $n^{if}p_{a}^{if}\gg n_{i}^{2}$. Therefore, Equation (1) can be simplified to Equation (2):

(2) $$R=\frac{1}{\frac{1}{{S_{n}p}_{a}^{if}}+\frac{1}{{S_{p}n}^{if}}}$$

Assuming that S_n_ ≈ S_p_ and the recombination of the heterojunction is cross recombination, when the electron affinity of CdS decreases from 4.2 eV to 3.9 eV (Cliff), the densities of electrons ($n^{if}$) and holes ($p_{a}^{if}$) at the heterojunction interface decrease (Table 2). According to Equation (2), it can be calculated that the interfacial recombination rate (R) of the CdS/CGS heterojunction decreases. This is consistent with the results shown in Figure 3. The change in J_SC_ of the device when the electron affinity (*χ*) of the CdS layer is reduced to 3.9 eV can be observed in Figure 3a. Figure 3a–d show that the bulk recombination (J_bulk-rec_) (0 V) under short-circuit conditions slightly decreases, leading to a decrease in total recombination (J_Tot-Rec_) (0 V). This may be the reason for the slight increase in J_SC_ when the electron affinity (*χ*) of CdS is reduced to 3.9 eV. In the case of FF ($FF=V_{Mpp}J_{Mpp}/(V_{OC}J_{SC})$), its value is related to the maximum power point value (V_MPP_ and J_MPP_). Figure 3a demonstrates that when the electron affinity decreases from 4.2 eV (Cliff) to 3.9 eV (Cliff), the V_MPP_ value at the maximum power point increases, while J_MPP_ changes slightly. Since the V_OC_ and V_MPP_ of the device increase, but J_MPP_ and J_SC_ change slightly, this may be the reason for the small change in FF of the device when the electron affinity is reduced to 3.9 eV (Cliff).

Based on the comprehensive analysis above, it can be seen that when the electron affinity of the CdS layer is reduced to 3.9 eV, the CdS/CGS heterojunction remains in the “Cliff” structure, but its intensity decreases. This is beneficial for reducing the interfacial recombination of the CdS/CGS heterojunction under high bias voltage, thus improving the *V*_OC_ of the device. At the same time, the weakening of the “Cliff” intensity also helps to reduce the bulk recombination of the device during short circuits, thereby improving the *J*_SC_ of the device. Although the weakening of the “Cliff” intensity has a relatively small impact on the *FF* value of the device, the performance of the device increases due to the increase in *V*_OC_ and *J*_SC_. Therefore, reducing the “Cliff” intensity of the CdS/CGS heterojunction is beneficial for improving the performance of the device.

### 3.2. The Impact of “Spike” Structure in CdS/CGS Heterojunction on Device Performance

When the electron affinity of the CdS layer is reduced to 3.5 eV, the CdS/CGS heterojunction is in the “Spike” structure (Figure 1b). Figure 2a–d show that when the heterojunction transitions to the “Spike” structure, the *V*_OC_ of the device increases significantly, the *J*_SC_ decreases slightly, the *FF* decreases significantly, and the device efficiency (*E*_ff_) decreases noticeably. To further understand the impact of structural changes in the CdS/CGS heterojunction on the performance of CGS devices, a detailed analysis is conducted in terms of the band structure and recombination mechanisms. As seen in Figure 3a–d, when the electron affinity of the CdS layer is reduced to 3.5 eV (Spike), the bulk recombination (*J*_bulk-rec_) (0 V) under short-circuit conditions increases, leading to an increase in the total recombination (*J*_Tot-Rec_) (0 V), which may be the reason for the decrease in *J*_SC_. The increase in bulk recombination (*J*_bulk-rec_) can be attributed to the formation of a barrier at the heterojunction interface, which hinders the transport of photo-generated carriers within the CGS absorber layer, as depicted in Figure 1. Additionally, Figure 3b indicates that the total recombination (*J*_Tot-Rec_) (1.4 V) further decreases at high bias voltage, when the electron affinity of the CdS layer is reduced to 3.5 eV (Spike). Figure 3b–d show that the reduction in interfacial recombination (*J*_jir-rec_) (1.4 V) is the primary reason for the decrease in total recombination (*J*_Tot-Rec_) (1.4 V). Therefore, the increase in *V*_OC_ may be related to the decrease in interfacial recombination (*J*_jir-rec_) (1.4 V). The calculation results of Equation (2) on the data in Table 2 further support the observation that the interfacial recombination at the CdS/CGS heterojunction is further reduced when the heterojunction is in the “Spike” structure. Regarding the impact of reducing the electron affinity of the CdS layer to 3.5 eV (Spike) on the *FF*, we analyzed the changes in the maximum power point (*V*_MPP_, *J*_MPP_) of the CGS device. As seen in Figure 3a, when the CdS/CGS heterojunction transitions to the “Spike” structure, the *V*_Mpp_ value of the device decreases while the *J*_MPP_ remains relatively unchanged. Therefore, when the electron affinity of the CdS layer is reduced to 3.5 eV, the decrease in the *FF* of the device may be attributed to the increase in *V*_OC_ and the decrease in the *V*_MPP_ value. It can be seen from Figure 3a–d that the decrease in the *V*_MPP_ may be related to a sharp increase in bulk recombination (*J*_bulk-Rec_) at this point. The increase in bulk recombination may be caused by the increased barrier at the heterojunction interface, which blocks the transportation of the photo-generated carrier of the CGS absorber.

Based on the above analysis, it can be seen that the “Spike” structure of the CdS/CGS heterojunction is beneficial for further reducing interfacial recombination at high bias voltage, which is conducive to improving the *V*_OC_ of the device. However, the “Spike” structure of the CdS/CGS heterojunction can lead to the formation of a barrier that hinders the transport of photo-generated carriers within the CGS absorber layer, resulting in increased bulk recombination. The decrease in the *R*_b_ observed in the Nyquist plot may also be attributed to an increase in bulk recombination, as depicted in Figure S1. This will in turn cause a decrease in *J*_SC_ and *FF*, thereby leading to a decline in device performance. Overall, the “Spike” structure of the heterojunction formed by the wide band gap CGS absorber layer and the buffer layer is not beneficial for improving the performance of the device.

## 4. Discussion

**Figure 4:**
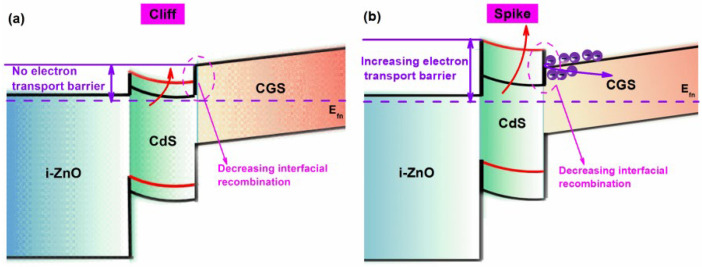
Schematic diagram of the energy band structure and photo-generated electron transport when the heterojunction is in (**a**) “Cliff” structure and (**b**) “Spike” structure.

Comparing the “Cliff” and “Spike” structures of the CGS/CdS heterojunction, it can be observed that when it is in the “Cliff” structure, reducing the amplitude of the “Cliff” is beneficial for decreasing the interfacial recombination of the heterojunction under high bias voltage, thereby promoting the enhancement of the device’s V_OC_ (Figure 4a). Simultaneously, as the CGS/CdS heterojunction is in the “Cliff” structure, no electronic barrier is formed at its interface. This will not hinder the transport of photo-generated carriers within the CGS absorber layer, resulting in minimal impact on the device’s FF and J_SC_ (Figure 4a). Therefore, adjusting the electron affinity of the buffer layer to weaken the “Cliff” degree is advantageous for enhancing device performance. However, the “Spike” structure of the CGS/CdS heterojunction is detrimental to device performance. This is primarily due to the fact that the “Spike” structure of the CGS/CdS heterojunction further reduces interfacial recombination and enhances the device’s V_OC_. However, a barrier at the interface of the heterojunction can be formed that hinders the transport of photo-generated carriers within the absorber layer. This can lead to increased bulk recombination and subsequently hamper the enhancement of the device’s J_SC_ and FF (Figure 4b). Consequently, when the CGS/CdS heterojunction is in the “Spike” structure, although the V_OC_ of the device may increase, the J_SC_ and FF decrease, resulting in no overall improvement in device performance. Additionally, through comparative analysis of the impact of the two heterojunction structures on the back recombination of the device, we have found that their influence on back recombination is relatively weak, as shown in Figure S2. The primary factor causing changes in device performance is the interfacial recombination and bulk recombination that result from variations in the heterojunction structure.

Based on the above analysis, it can be inferred that the use of adjustable gap buffer layers (such as Zn(O,S) and Zn(Mg,O)) can enhance the performance of wide band gap CIGS solar cell devices. If the heterojunction structure formed between the wide band gap CIGS absorber layer and the buffer layer is in a weak “Cliff” structure, it is beneficial for reducing interfacial recombination and improving the V_OC_ of the device. A barrier that hinders photo-generated electron transport in this weak “Cliff” structure will not be formed, thereby avoiding a significant negative impact on the FF and J_SC_ of the device. This simulation result concurs with the experimental findings of K. T. Ramakrishna Reddy and S. Sharbati et al., who indicated that the “Spike” structure does not help to enhance the performance of wide-gap CGS solar cells [19,20]. Additionally, we conducted a simulation to investigate the impact of the heterojunction structure on the performance of wide band gap CGS solar cells, considering different front surface recombination velocities (*S*_p/n_), absorber and buffer doping densities, carrier lifetimes, and external quantum efficiencies (EQEs) (Figures S3–S8). The results indicate that although the device performance exhibits different values in these various scenarios, they all basically arrive at the same conclusion as mentioned above.

## 5. Conclusions

In this paper, CGS solar cells are selected as research samples to deeply explore the influence of “Cliff” and “Spike” heterojunction structures of CGS/CdS on the performance of wide band gap CIGS devices. According to the investigation, it is found that reducing the “Cliff” degree of the CdS/CGS heterojunction (such as when the electron affinity of the CdS buffer layer is 3.9 eV) helps minimize interfacial recombination at the heterojunction. This is beneficial for improving the *V*_OC_, which is the primary reason for enhancing device performance. However, when the heterojunction transitions to the “Spike” structure (such as when the electron affinity of the CdS buffer layer is 3.5 eV), interfacial recombination decreases further, but an electronic transportation barrier is formed at the heterojunction interface. This leads to an improvement in *V*_OC_ but a decrease in *J*_SC_ and *FF*, ultimately resulting in a decrease in the overall device performance. Therefore, the “Spike” structure heterojunction is not conducive to improving the performance of wide band gap CIGS solar cell devices. Instead, to achieve optimal efficiency in wide band gap CIGS solar cells, a weak “Cliff” structure should be selected as the ideal heterojunction structure. The aforementioned research results offer highly valuable insights and serve as a reference for exploring the impact of heterojunction structures on enhancing the performance of wide band gap CIGS solar cell devices.

To simplify simulation, this article analyzes the impact of different heterojunction structures on wide band gap CIGS solar cells, utilizing the most commonly used CdS buffer layer with a constant band gap. Since buffer layers with adjustable band gaps (such as Zn(O,S) and Zn(Mg,O)) are more conducive to enhancing the performance of CIGS solar cell devices, our research findings offer valuable insights. These findings also serve as a reference for improving the performance of wide band gap CIGS solar cell devices through the use of buffer layers with adjustable band gaps.

## Figures and Tables

**Table 1:** Material parameters used in the simulation [25,26,27].

Layer	Window	Window	Buffer	Absorber
parameter	Al-ZnO	i-ZnO	CdS	CGS
*E*_g_ (eV)	3.5	3.5	2.4	1.68
*N*_D_ (cm^−3^)	10^18^	10^17^	10^17^	10^16^
*χ* (eV)	4.42	4.42	variable	3.68
Thickness	350 nm	50 nm	50 nm	2 μm
ε/ε_0_	10	10	10	13.6
*N*_C_ [cm^−3^]	1 × 10^18^	1 × 10^18^	1 × 10^18^	1 × 10^18^
*N*_V_ [cm^−3^]	1 × 10^19^	1 × 10^19^	1 × 10^19^	1.8 × 10^19^
*μ*_p_ [cm^2^/(Vs)]	100	100	100	100
*μ*_n_ [cm^2^/(Vs)]	25	25	25	25
ν_th,p_ [cm/s]	1 × 10^7^	1 × 10^7^	1 × 10^7^	1 × 10^7^ [25]
ν_th_,_n_ [cm/s]	1 × 10^7^	1 × 10^7^	1 × 10^7^	1 × 10^7^ [25]

**Table 2:** Concentrations of holes on the CGS side of the heterojunction ($p_{a}^{if}$) and concentrations of electrons at the heterojunction interface (n^if^) with different affinity potentials of the buffer layer.

Interface (CGS/CdS)	$n^{if}$ (cm^−3^)	$p_{a}^{if}$ (cm^−3^)
CdS (*χ* = 4.2)	3.58 × 10^15^	39.6
CdS (*χ* = 3.9)	1.87 × 10^11^	7.07
CdS (*χ* = 3.5)	3.6 × 10^3^	7.07

## Data Availability

The original contributions presented in the study are included in the article/Supplementary Material, further inquiries can be directed to the corresponding author.

## Supplementary Materials

The following supporting information can be downloaded at: https://www.mdpi.com/article/10.3390/ma17215199/s1.
