# Supplementary material for: Optimizing Wide Band Gap Cu(In,Ga)Se2 Solar Cell Performance: Investigating the Impact of “Cliff” and “Spike” Heterostructures
Source: Materials (Basel). 2024 Oct 25;17(21):5199. doi: 10.3390/ma17215199 (PMC11547025; doi:10.3390/ma17215199)
Supplement: Supplementary file 1 [file materials-17-05199-s001.zip › materials-3220003-supplementary.pdf]

Supplementary

1. SCAPS simulation program

1.1 Overview of SCAPS Software

SCAPS is a professional solar cell simulation software that predicts the photoelectric conversion performance of solar cells based on physical models. It considers various parameters of solar cells, such as material properties, structural design, and illumination conditions, to provide accurate simulation results.

1.2 Simulation Settings and Parameters

In our study, we used the SCAPS software with the following key parameters

Mesh size/density: The cell area of the solar cells is 1.0 cm^2, the doping concentration of Al-ZnO, i-ZnO, CdS and CGS layers are 10^{18} cm^{-3}, 10^{16} cm^{-3}, 10^{17} cm^{-3}, 10^{16} cm^{-3} respectively.

Material parameters: These parameters can be seen in Table 1 of the manuscript.

Table S1: material parameters used in simulation[25-27]

Layer	window	window	Buffer	Absorber
parameter	Al-ZnO	i-ZnO	CdS	CGS
E_g (eV)	3.5	3.5	2.4	1.68
N_D (cm^{-3})	10^{18}	10^{16}	10^{17}	10^{16}
χ (eV)	4.42	4.42	variable	3.68
Thickness	350 nm	50 nm	50 nm	2 μm
$\varepsilon/\varepsilon_0$	10	10	10	13.6
N_C[cm^{-3}]	1×10^{18}	1×10^{18}	1×10^{18}	1×10^{18}
N_V[cm^{-3}]	1×10^{19}	1×10^{19}	1×10^{19}	1.8×10^{19}
μ_p[$cm^2/(Vs)$]	100	100	100	100
μ_n[$cm^2/(Vs)$]	25	25	25	25
$v_{th,p}$[cm/s]	1×10^7	1×10^7	1×10^7	1×10^7[25]
$v_{th,n}$[cm/s]	1×10^7	1×10^7	1×10^7	1×10^7[25]

Structural design: The structure of the CGS solar cells is Al-ZnO/i-ZnO/CdS/CGS

Illumination conditions: The illumination is provided by a one-sun (1000 W/m^2) source with an air mass of 1.5, using the global spectrum (AM1.5).

1.3 Sensitivity Analysis:

To assess the reliability of the simulation results, we conducted a sensitivity analysis. The main focus of this paper is to alter the heterojunction structure of the CdS/CGS layer by modifying the electron affinity of the CdS layer, thereby influencing the performance of the device. We investigated the impact of external circuit load on the simulation results. The results indicated that changes in external circuit load within a reasonable range had a minor impact on the performance of the

solar cell, thus validating the stability of our simulation results.

1.4 Simulation Results and Interpretation

Cliff Structure Influence:

When the CdS/CGS hetero-junction exhibits a reduced "Cliff" degree (such as the electron affinity of the CdS buffer layer of 3.9 eV), it helps minimize interfacial recombination at the hetero-junction. This minimization of interfacial recombination is beneficial for improving the open-circuit voltage (V_{OC}) of the CIGS solar cell.The improvement in V_{OC} is identified as the primary reason for enhancing the overall device performance in this scenario.

Spike Structure Influence:

As the hetero-junction transitions to a "Spike" structure (such as the electron affinity of the CdS buffer layer of 3.5 eV), interfacial recombination further decreases. However, this transition also leads to the formation of an electronic transportation barrier at the hetero-junction interface. Although the V_{OC} will be improved, there will be a notable decrease in both the short-circuit current density (J_{SC}) and the fill factor (*FF*).These decreases ultimately result in a decline in the overall device performance when compared to the "Cliff" structure with a reduced degree.

Optimal Hetero-Junction Structure:

Based on the simulation results, a weak "Cliff" structure is identified as the ideal hetero-junction structure for achieving optimal efficiency in wide bandgap CIGS solar cells. This structure minimizes interfacial recombination while avoiding the formation of electronic transportation barriers, thereby enhancing both V_{OC} and overall device performance.

1.5 Conclusion

In conclusion, our SCAPS simulation results provide compelling evidence to support our research conclusions.

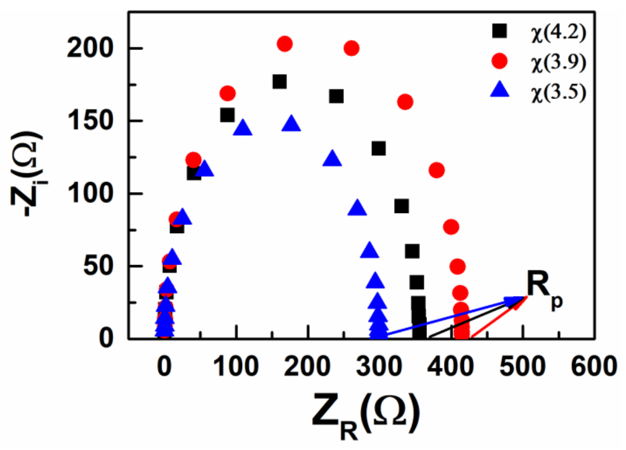

Figure S1 Nyquist plots of the different hetero-junctions, Cliff (χ=4.2 , 3.9) and Spike (χ=3.5), R_p is recombination resistance.

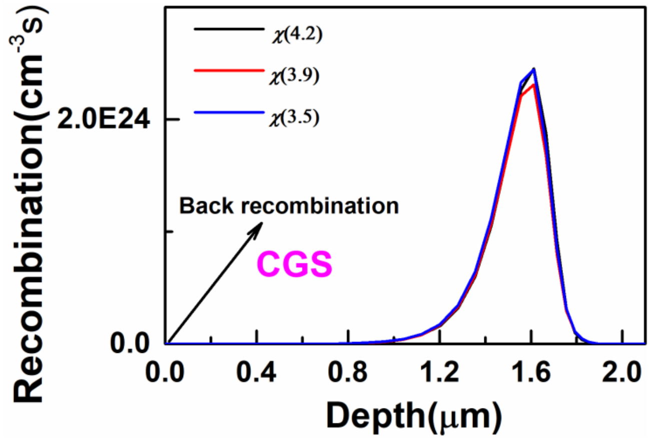

Figure S2 Recombination profile with solar cell depth

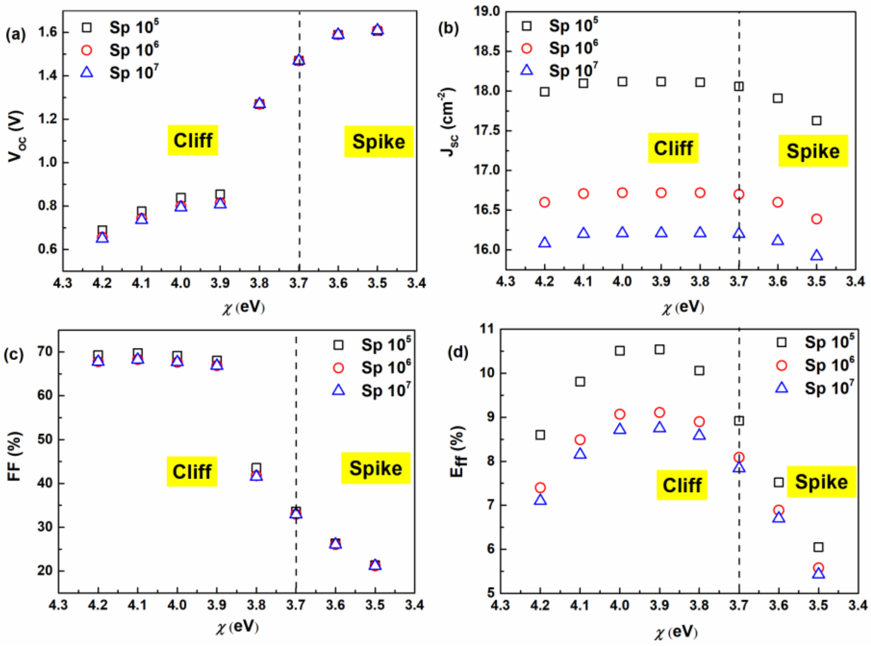

Figure S3 The influence of the change of electronic affinity of CdS layer (χ) on the wide band gap CGS solar cells performance with different surface recombination velocity (S_p). (a) V_{OC} (b)J_{SC}, (c)*FF* and (d) E_{ff}

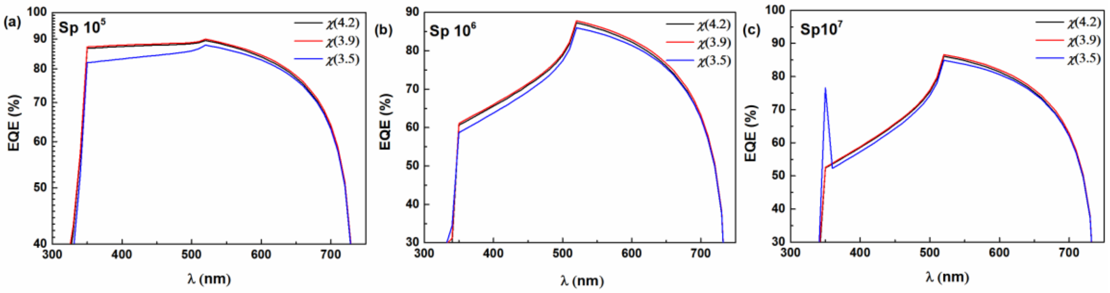

Figure S4 The influence of the change of electronic affinity of CdS layer (χ) on the wide band gap CGS solar cells external quantum efficiency (EQE) with different surface recombination velocity (a) S_p=10^5 cm/s (b)S_p=10^6 cm/s, (c)S_p=10^7 cm/s

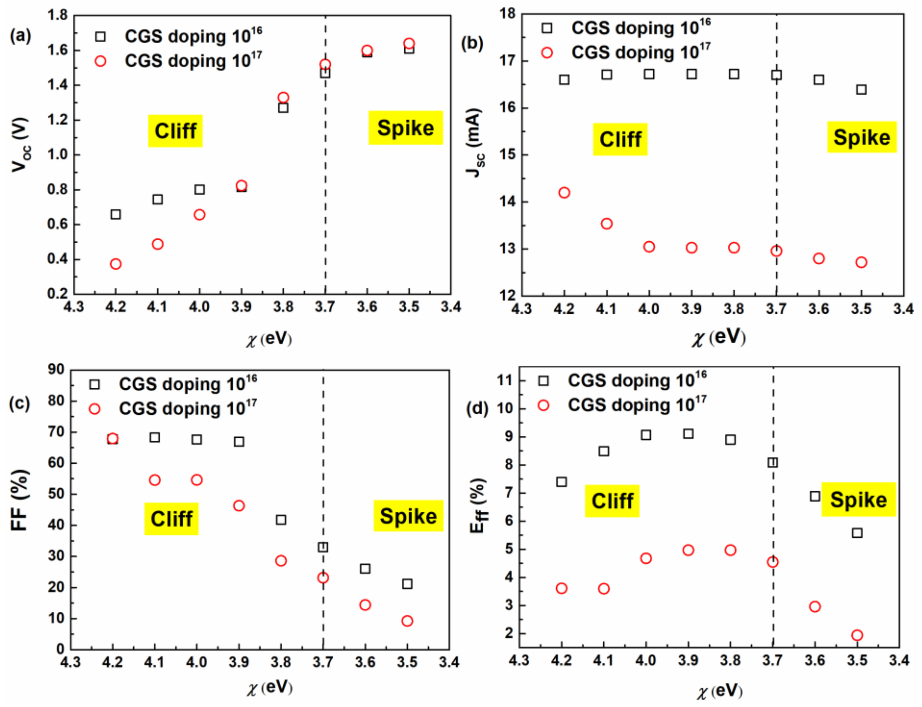

Figure S5 The influence of the change of electronic affinity of CdS layer (χ) on the wide band gap CGS solar cells performance with different CGS doping concentration. (a) V_{OC} (b)J_{SC}, (c)FF and (d) E_{ff}

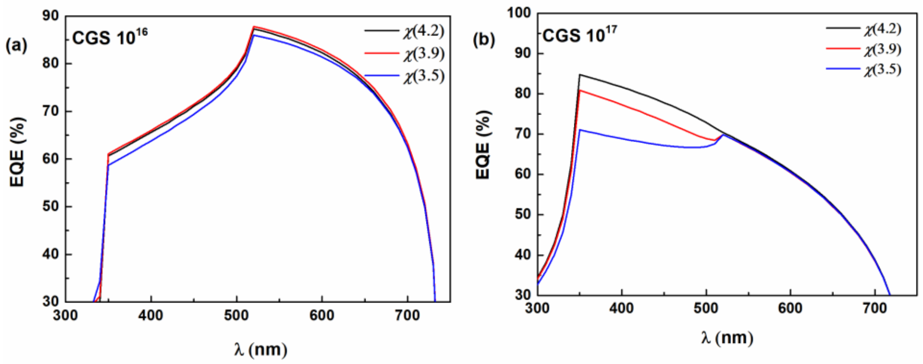

Figure S6 The influence of the change of electronic affinity of CdS layer (χ) on the wide band gap CGS solar cells external quantum efficiency (EQE) with different doping concentration (N_A) of CGS layer (a) N_A=10^{16} cm^{-3} (b) N_A=10^{17} cm^{-3}

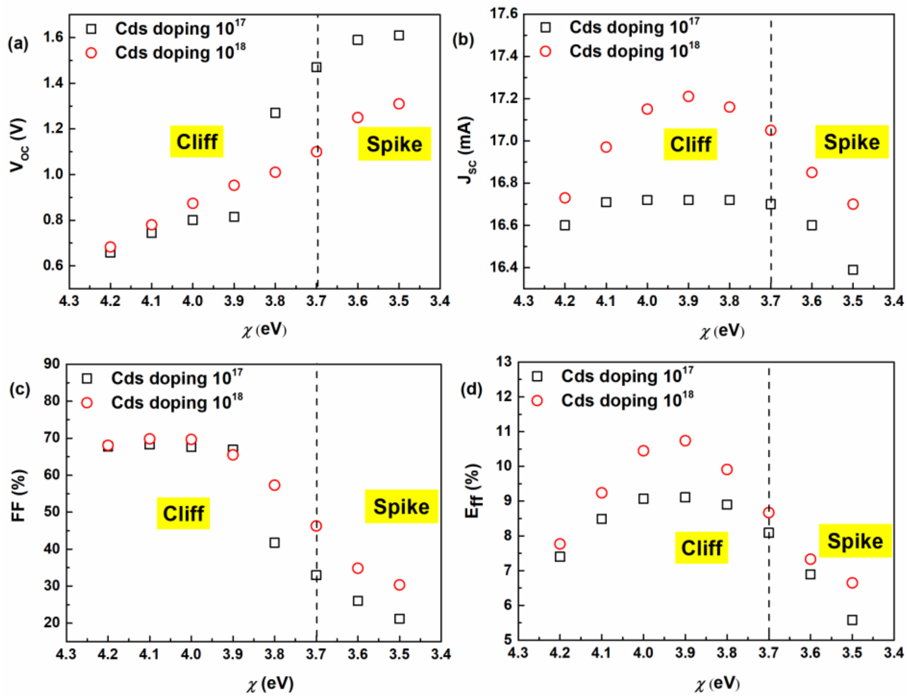

Figure S7 The influence of the change of electronic affinity of CdS layer (χ) on the wide band gap CGS solar cells performance with different CdS layer doping concentration. (a) V_{OC} (b)J_{SC}, (c)FF and (d) E_{ff}

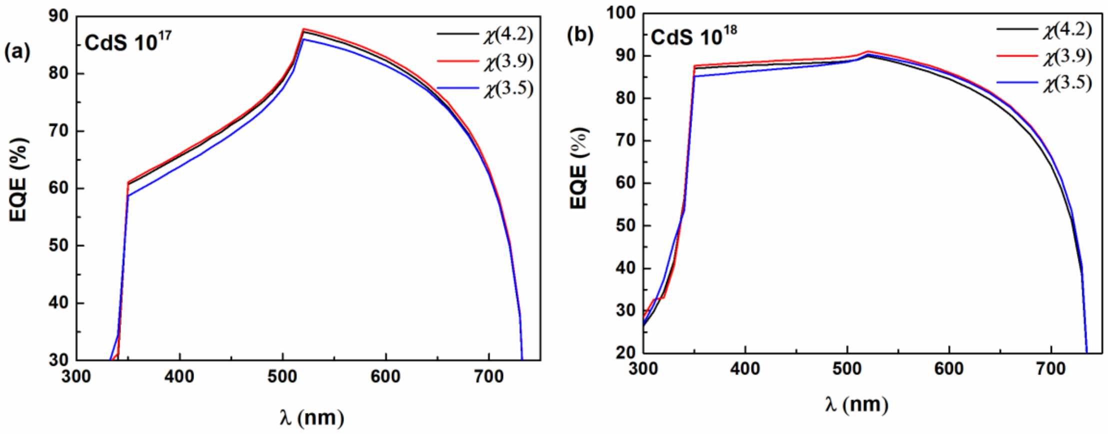

Figure S8 The influence of the change of electronic affinity of CdS layer (χ) on the wide band gap CGS solar cells external quantum efficiency (EQE) with different doping concentration (N_D) of CdS layer (a) $N_D=10^{17}$ cm^{-3} (b) $N_A=10^{18}$ cm^{-3}
